# Molecular cross-talk among human intestinal bifidobacteria as explored by a human gut model

**DOI:** 10.3389/fmicb.2024.1435960

**Published:** 2024-09-09

**Authors:** Sonia Mirjam Rizzo, Giulia Alessandri, Chiara Tarracchini, Massimiliano G. Bianchi, Alice Viappiani, Leonardo Mancabelli, Gabriele Andrea Lugli, Christian Milani, Ovidio Bussolati, Douwe van Sinderen, Marco Ventura, Francesca Turroni

**Affiliations:** ^1^Laboratory of Probiogenomics, Department of Chemistry, Life Sciences, and Environmental Sustainability, University of Parma, Parma, Italy; ^2^Laboratory of General Pathology, Department of Medicine and Surgery, University of Parma, Parma, Italy; ^3^Microbiome Research Hub, University of Parma, Parma, Italy; ^4^GenProbio Srl, Parma, Italy; ^5^Department of Medicine and Surgery, University of Parma, Parma, Italy; ^6^APC Microbiome Institute and School of Microbiology, Bioscience Institute, National University of Ireland, Cork, Ireland

**Keywords:** *Bifidobacterium*, probiotic, co-association, gut microbiota, gut microbiome, co-culture, metatranscriptomics

## Abstract

Bifidobacteria are well known as common and abundant colonizers of the human gut and are able to exert multiple beneficial effects on their host, although the cooperative and competitive relationships that may occur among bifidobacterial strains are still poorly investigated. Therefore, to dissect possible molecular interactions among bifidobacterial species that typically colonize the human gut, three previously identified bifidobacterial prototypes, i.e., *B. bifidum* PRL2010, *B. breve* PRL2012, and *B. longum* PRL2022 were cultivated individually as well as in bi- and tri-association in a human gut-simulating medium. Transcriptomic analyses of these co-associations revealed up-regulation of genes predicted to be involved in the production of extracellular structures including pili (i.e., flp pilus assembly TadE protein gene), exopolysaccharides (i.e., GtrA family protein gene) and teichoic acids (i.e., ABC transporter permease), along with carbohydrate, amino acid and vitamin metabolism-related genes (i.e., exo-alpha-sialidase; beta-galactosidase and pyridoxamine kinase), suggesting that co-cultivation of bifidobacteria induces a response, in individual bifidobacterial strains, aimed at enhancing their proliferation and survival, as well as their ability to cooperate with their host to promote their persistence. Furthermore, exposure of the selected prototypes to human cell line monolayers unveiled the ability of the bifidobacterial tri-association to communicate with their host by increasing the expression of genes involved in adherence to/interaction with intestinal human cells. Lastly, bifidobacterial tri-association promoted the transcriptional upregulation of genes responsible for maintaining the integrity and homeostasis of the intestinal epithelial barrier.

## Introduction

The interactions among bacteria inhabiting the human gut impact host health by altering human metabolism and influencing the presence and virulence of pathogens (Tojo et al., 2014; Milani et al., 2017). Notably, metabolic interactions between particular gut microbiota members may encompass synergistic activities or antagonistic occurrences, such as those observed between transient and indigenous gut microorganisms (Robles Alonso and Guarner, 2013; Rescigno, 2014). In this context, recent scientific research has included various molecular investigations pertaining to members of the genus *Bifidobacterium* (Margolles et al., 2003; Turroni et al., 2009; Bottacini et al., 2010; Turroni et al., 2012; Laursen et al., 2021; Altaib et al., 2022), which are considered early colonizers of the human gut. Bifidobacteria appear to have a symbiotic relationship with their host, where the latter profits through various purported health benefits, including defense against pathogens, reduction of gut inflammation, immune system modulation, and strengthening the protective mucus layer that coats the intestinal lining. Additionally, bifidobacteria metabolize a large variety of non-digestible glycans and produce various health-related metabolites, including short-chain fatty acids, vitamins, and polyphenols (Ventura et al., 2012; Schiavi et al., 2016; Turroni et al., 2018a; Blanco et al., 2020; Choi et al., 2021; Quintanilha et al., 2022). Bifidobacteria are highly prevalent and abundant inhabitants of the infant intestine, though their presence decreases after weaning; nonetheless, they persist in the adult gut (at 1–5% relative abundance), levels that are believed to be sufficient to provide their host with the aforementioned advantages (Avershina et al., 2013; Milani et al., 2015b; Avershina et al., 2016). Specifically, host sex-related persistence of strains belonging to common, maternally inherited bifidobacterial species, including *Bifidobacterium longum* subsp. *longum* and *Bifidobacterium bifidum*, was observed based on their ability to degrade host-derived glycans (Tarracchini et al., 2023; Rizzo et al., 2024). Members of the species *B. bifidum*, *B. longum*, and *Bifidobacterium breve* are not only the first commonly encountered species colonizing the gut, but they also exert beneficial influence on the gut microbial ecosystem and toward the epithelial host cells (Hougee et al., 2010; Bozzi Cionci et al., 2018; Tanno et al., 2019; Yan et al., 2019; Turroni et al., 2020). In this context, it has been demonstrated that bifidobacteria interact with each other through cross-feeding relationships, thereby enhancing their fitness within the human intestinal environment and exerting beneficial effects on their host (Turroni et al., 2014b; Riviere et al., 2016; Turroni et al., 2016; Turroni et al., 2018b).

Thus, to dissect and investigate putative molecular synergies between bifidobacterial strains and to determine whether their interactions may enhance their host colonization and promote beneficial health effects, three bifidobacterial strains, i.e., *B. bifidum* PRL2010, *B. longum* PRL2022, *B. breve* PRL2012 were selected. These strains were previously identified as prototypes of the human gut microbiota based on functional and genomic surveys (Fontana et al., 2022; Alessandri et al., 2023; Argentini et al., 2024). These strains were grown in bi-and tri-association in a human intestinal environment-simulating medium (Alessandri et al., 2022) and their molecular cross-talk activities were investigated through a transcriptomic analysis. Moreover, an *in vitro* experiment involving Caco-2/HT-29-MTX cells was employed to evaluate whether the association of the three selected bifidobacterial strains when in contact with human cell intestinal monolayer may impact both bifidobacterial and human cell line transcriptomes.

## Materials and methods

### Bifidobacterial bi-and tri-association

To evaluate whether or not the co-association of the selected bifidobacterial strains (*B. bifidum* PRL2010 and *B. longum* PRL2022 were isolated from human infant/adult feces respectively, while *B. breve* PRL2012 was isolated from human milk) impacts their gene expression, the three strains were cultivated in mono-, bi-and tri-associations in a human colon environment-simulating growth medium (IGSM) (Alessandri et al., 2022) supplemented with 0.05% (wt/vol) L-cysteine hydrochloride under anaerobic conditions (2.99% H_2_, 17.01% CO_2_, and 80% N_2_). The IGSM contains various sources of carbohydrates, i.e., inulin (0.2 g/L), pectin (0.2 g/L), arabinogalactan (0.2 g/L), xylan (0.2 g/L), lactose (0.2 g/L), mucin (3 g/L) and starch (3 g/L). Specifically, viable cells were inoculated in 30 mL of freshly prepared IGSM medium. The bifidobacterial cells were enumerated by using the Thoma cell counting chamber (Herka). Cells were inoculated approximately to 1×10^6^ cells /mL. After inoculation, growth was monitored until the exponential phase was reached (around 1×10^8^ cells/mL), then cells were harvested by centrifugation at 6000 rpm for 5 min. Bacterial cells were stored at −80°C until they were processed for RNA extraction. Growth assays were carried out in triplicate.

### DNA extraction and qPCR analysis

An aliquot of each condition, i.e., mono-culture, bi-association and tri-association was subjected to DNA extraction using the QIAmp DNA Stool Mini kit following the manufacturer’s instructions (Qiagen, Germany). Subsequently, to evaluate bifidobacterial cell numbers in each sample, a quantitative PCR (qPCR) was performed using *B. bifidum* PRL2010, *B. breve* PRL2012 and *B. longum* PRL2022-specific primers listed in the Supplementary Table S14. qPCR was performed using the PowerUp SYBR Green Master Mix (Applied Biosystem, United States) on a CFX96 system (BioRad, CA, United States) following previously described protocols (Milani et al., 2015a). PCR products were detected with SYBR green fluorescent dye and amplified according to the following protocol: one cycle of 95°C for 2 min, followed by 40 cycles of 95°C for 15 s, 58°C for 15 s, and 72°C for 1 min. The melting temperature was 56°C for *B. longum* PRL2022. In each run, negative controls (no DNA) were included. A standard curve was generated using the CFX96 software (BioRad). In detail, a known DNA standard sample for each species was selected, quantified, and serially diluted to obtain the number of copies of double-stranded DNA per μL ranging from 10^3^ to 10^9^. The standard curve was then automatically generated by the software.

### Human cell line trials

Caco-2 cells, derived from a colorectal adenocarcinoma of a human male donor (purchased from ATCC), and HT-29-MTX, a human colon carcinoma-derived mucin-secreting goblet cell line from a female donor (kindly provided by prof. Antonietta Baldi, University of Milan) were cultured in Minimum Essential Medium (MEM) and Dulbecco’s Modified Eagle’s medium (DMEM) with high glucose (4.5 g/L) and sodium pyruvate (10 mM), as previously described (Bianchi et al., 2019). Both media were supplemented with 10% Fetal Bovine Serum (FBS), 2 mM glutamine, 100 μg/mL streptomycin, and 100 U/mL penicillin. Cultures were maintained at 37°C in a 5% CO_2_ humidified atmosphere in 10-cm dishes and passaged three times a week. Subsequently, a mixed suspension of Caco-2 and HT-29-MTX cells (7, 3) was seeded in DMEM + FBS at a density of ≈10^5^ cells/cm^2^ into cell culture inserts with membrane filters (pore size 0.4 μm) for Falcon 24-well-multitrays (Becton, Dickinson & Company, Franklin Lakes, NJ, United States). Cells were grown for 21 days until a tight monolayer was formed (TEER >600 Ω x cm^2^) with medium replacement every 3 days.

### Co-cultures of human cell monolayers and bifidobacteria

After 21 days from seeding, the culture medium of the 24-well plates was replaced with fresh, antibiotic-free DMEM. Subsequently, 400 μL of bifidobacterial cells with a final concentration of ≈10^8^ cells/ml were inoculated on the Caco-2/HT-29-MTX cell monolayers, as previously described (Serafini et al., 2013; Rizzo et al., 2023). The 24-well plates were then incubated at 5% CO_2_ at 37°C. After 4 h of incubation, the non-adherent bacterial cells were washed using 500 μL of PBS (Phosphate Buffered Saline), then the adherent bacterial cells were gently detached from the human cells using a pipette with 500 μL of RNA later and stored at −80°C until processing. For this experiment, the three bifidobacterial strains were grown in the IGSM medium in anaerobic conditions at 37°C. Once the exponential growth phase was reached, bifidobacterial cells were enumerated by using the Thoma cell counting chamber (Herka). The bacterial cells were diluted to reach a final concentration of 10^8^ cells/mL in 400 μL, washed in the same volume of PBS, and then resuspended in 400 μL of antibiotic-free DMEM. At the end the bacterial cells were added to the cell monolayers. Bacterial studies were performed either with single strains (used as sample control) or with a mixture of the three strains and added to the human cell monolayers. All experiments were performed in triplicate, with three technical replicates for each biological replicate.

### Prokaryotic RNA extraction and sequencing

Total RNA from each bifidobacterial culture was isolated as previously described (Turroni et al., 2010b). Briefly, cell pellets were resuspended in 1 mL of QIAZOL (Qiagen, United Kingdom) and placed in a tube containing 0.8 g of glass beads (diameter, 106 μm; Sigma). Cells were lysed by alternating 2 min of agitating the mix on a bead beater with 2 min of static cooling on ice. The mixture was then centrifuged at 12,000 rpm for 15 min, and the RNA-containing sample was recovered from the upper phase. The latter was further processed using the RNeasy mini kit (Qiagen, Germany) according to the manufacturer’s instructions. The quality of the RNA was verified employing a Tape station 2200 (Agilent Technologies, United States). RNA concentration and purity were evaluated using a spectrophotometer (Eppendorf, Germany). For RNA sequencing, from 100 ng to 1 μg of extracted RNA was treated to remove rRNA by employing QIAseq FastSelect – 5S/16S/23S following the manufacturer’s instructions (Qiagen, Germany). RNA yield following rRNA depletion was checked by the use of a Tape station 2200 (Agilent Technologies, USA). Subsequently, a whole transcriptome library was constructed using the TruSeq Standard mRNA preparation kit (Illumina, San Diego, United States). Samples were processed using a NextSeq high output v2.5 kit (150 cycles, single end) and sequenced (Illumina) according to the technical support guide. The obtained reads were filtered to remove low-quality reads (minimum mean quality 20 and minimum length 150 bp) as well as any remaining ribosomal locus-encompassing reads using the METAnnotatorX2 (Milani et al., 2021). Subsequently, the retained reads were aligned to the specific reference genome of each employed bifidobacterial strain through Bowtie2 software (Langdon, 2015). Quantification of reads mapped to individual transcripts was achieved through htseq-counts script of HTSeq software in “union” mode (Anders et al., 2015). Raw counts were then normalized using CPM (Counts per million mapped reads) for filtering genes with low counts (CPM <1) and TMM (Trimmed Mean of M-Values) for statistically robust differential gene expression analysis through the EdgeR package (Robinson et al., 2010). Evaluation of expression differences was calculated for each gene as log2 fold change (logFC) of average expression between the control (the single strain) and “treated” samples (strains all together and in pairs). Additionally, for each comparison, a Volcano plot was created to simultaneously visualize expression changes (log fold change) and their statistical significance (*p*-value).

### Eukaryotic RNA extraction and analyses

Total RNA from human cells was isolated using a previously described method (Alessandri et al., 2023). Briefly, total RNA from human cell lines was extracted by adding 350 μL of RLT buffer from the RNeasy Mini Kit (Qiagen, Germany) and following the manufacturer’s instructions, then the RNA concentration and purity were evaluated using a spectrophotometer (Eppendorf, Germany). Reverse transcription to cDNA was conducted utilizing the iScript Select cDNA synthesis kit (Bio-Rad Laboratories), employing the following thermal cycle: 5 min at 25°C, followed by 30 min at 45°C, and concluding with 8 min at 85°C. The mRNA expression levels were assessed using SYBR green technology in quantitative Real-Time PCR (Biorad) executed on a Bio-RAD CFX96 system. Gene expression was standardized in relation to a housekeeping gene (*atp*D), as previously outlined (Turroni et al., 2011a). The primers used are listed in the Supplementary Table S14.

### Statistical analysis

For differential gene expression analysis, EdgeR package was used to estimate the statistical significance of differences between fold changes as the False Discovery Rate (FDR).

### Data availability

Raw sequences of RNA sequencing data are available in the SRA database with accession number PRJNA1108206.

## Results and discussion

### Effects of bifidobacterial prototype co-associations on transcriptomes

To evaluate whether particular co-associations of *B. bifidum* PRL2010, *B. longum* subsp. *longum* PRL2022, and *B. breve* PRL2012 strains induce a modification in the corresponding bifidobacterial transcriptomes, these bacteria were grown individually (mono-association), as well as in bi-and tri-associations, in a human gut environment-simulating culture medium. Alterations in gene expression in the bi-associations and tri-association compared to the single strain cultivation were evaluated through RNA sequencing. The latter generated a total of 2,379,454 quality-filtered reads, with an average of 67,984 reads per sample (Supplementary Table S1). In this context, only genes showing a fold-change in transcription of ≥2 in combination with a *p*-value of ≤0.05 calculated through correction for multiple comparisons using the False Discovery Rate (FDR) procedure were considered as significantly differentially transcribed between the bi-and tri-associations when compared to mono-associations. Specifically, the above-mentioned species were chosen due to the fact that they colonize the host throughout their entire lifespan. These specific strains were selected as prototype of the species they belong to as previously demonstrated through ecological and phylogenetic approaches (Turroni et al., 2011b; Turroni et al., 2012; Derrien et al., 2022; Turroni et al., 2022; Argentini et al., 2024).

**Figure 1:**
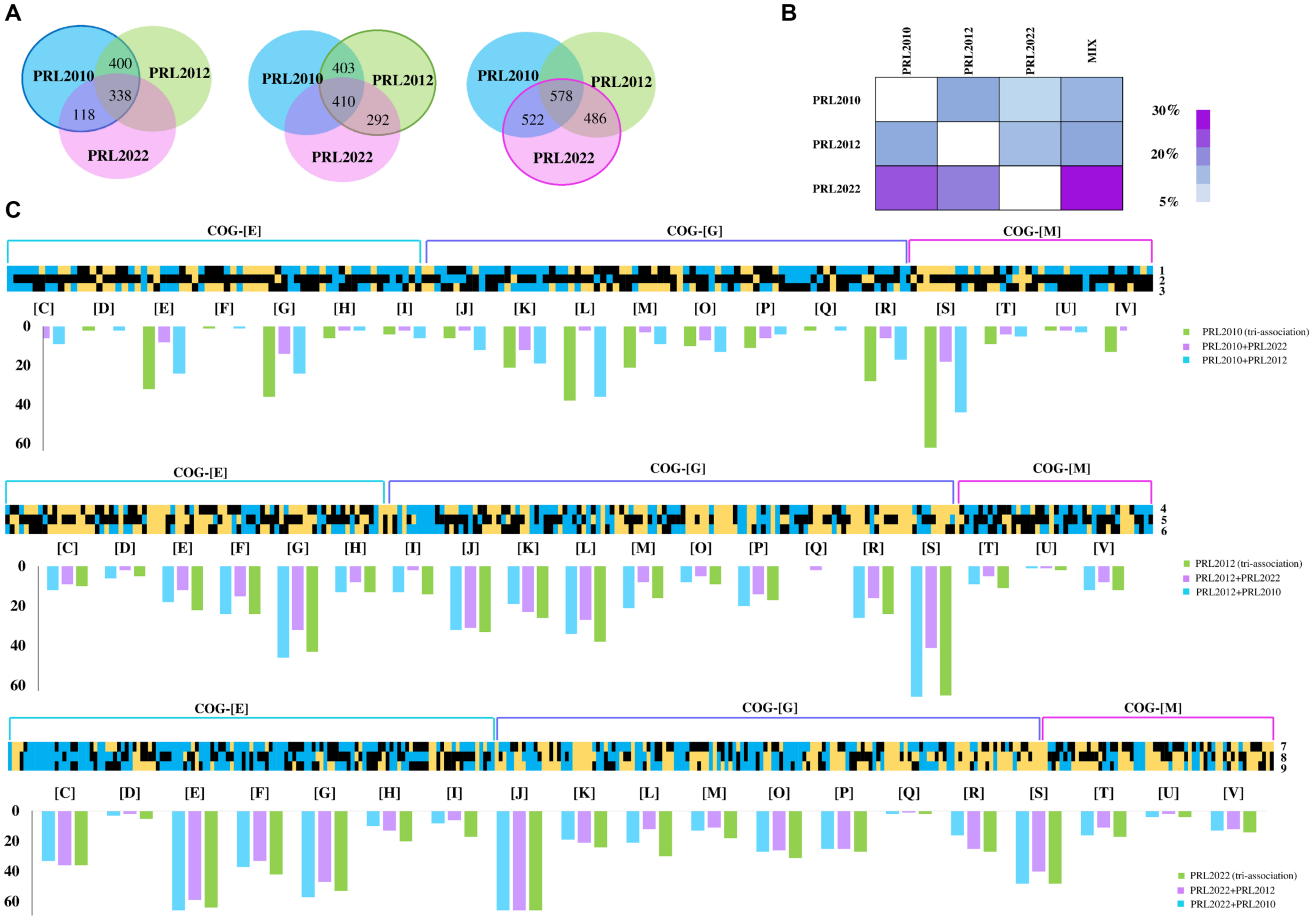
Transcriptome analyses of co-cultivated bifidobacterial strains. Panel **(A)** depicts the Venn diagrams representing the up-regulated genes in *B. bifidum* PRL2010, *B. breve* PRL2012, and *B. longum* PRL2022, respectively, in bi-and tri-association. Panel **(B)** reports on the cross-talk index of each bifidobacterial strain for all tested conditions. Panel **(C)** shows the *B. bifidum* PRL2010, *B. breve* PRL2012, and *B. longum* PRL2022 differentially expressed genes by transcriptome analysis in response to bi-and tri-associations. Each heat map displays fold change in gene expression for *B. bifidum* PRL2010, *B. breve* PRL2012, and *B. longum* PRL2022 according to the condition indicated by the number reported on the right of the heat maps. The numbers next to each individual column of the heat map correspond to the various associations of bifidobacteria: 1, *B. bifidum* PRL2010 + *B. breve* PRL2012; 2, *B. bifidum* PRL2010 + *B. longum* PRL2022; 3, *B. bifidum* PRL2010 (tri-association); 4, *B. breve* PRL2012 + *B. bifidum* PRL2010; 5, *B. breve* PRL2012 + *B. longum* PRL2022; 6, *B. breve* PRL2012 (tri-association); 7, *B. longum* PRL2022 + *B. bifidum* PRL2010; 8, *B. longum* PRL2022 + *B. breve* PRL2012; 9, *B. longum* PRL2022 (tri-association). The heat maps solely report on transcriptional differences of genes belonging to the COG-E, G and M categories. The bar plots under each heat map show the functional annotation of significantly differentially transcribed genes of bi-and tri-association separated according to the cluster of orthologous gene (COG) categories they belong to. Each COG family is identified by one-letter abbreviations: C, energy production and conversion; D, cell cycle control and mitosis; E, amino acid metabolism and transport; F, nucleotide metabolism and transport; G, carbohydrate metabolism and transport; H, coenzyme metabolism; I, lipid transport and metabolism; J, translation; K, transcription; L, replication and repair; M, cell wall/membrane/envelop biogenesis; N, cell motility; O, post-translational modification, protein turnover, chaperone functions; P, inorganic ion transport and metabolism; Q, secondary structure; R, general functional prediction only; S, function unknown; T, signal transduction; U, intracellular trafficking and secretion; V, defense mechanisms; Z, cytoskeleton.

Among the considered co-associations, in-depth insights into those genes with significantly altered transcription revealed that the highest number of up-regulated genes in *B. breve* PRL2012 and *B. longum* subsp. *longum* PRL2022 was observed when both were cultured in tri-associations. Analysis of *B. breve* PRL2012 demonstrated an up-regulation of 410 genes, while *B. longum* PRL2022 up-regulated 578 genes. However, regarding *B. bifidum* PRL2010 the highest up-regulation was demonstrated to be 400 genes, when co-cultured with *B. breve* PRL2012 (Figure 1A; Supplementary Tables S2–S10). Therefore, while the tri-association seemed to better stimulate cross-talk between *B. longum* PRL2022 and *B. breve* PRL2012, the presence of the single *B. breve* PRL2012 strain appeared to induce the most relevant impact on the transcriptome of *B. bifidum* PRL2010, with the number of *B. bifidum* PRL2010 up-regulated genes in the tri-association is slightly lower (338) (Figure 1A). This observation was further confirmed by the assessment of the cross-talk index, calculated for each bi-association and for the tri-association as the ratio between the number of over-expressed genes respect to the total gene repertoire for each genome, as previously described (Turroni et al., 2016; Figure 1B). In detail, the determination of the cross-talk index emphasized that the highest modulation of *B. bifidum* PRL2010 gene expression was achieved when *B. breve* PRL2012, regardless of the bi-or tri-associations, was present in the co-cultivation, suggesting a higher response of *B. bifidum* PRL2010 toward the interaction with *B. breve* PRL2012 (Figure 1B), reflecting also the frequent co-occurrence of these bacteria in the same ecological niche, i.e., the infant gut (Turroni et al., 2015). In this regard, it has been demonstrated by previous studies that strains of *B. bifidum* and *B. breve* generally establish an intimate trophic dialog based on metabolic cross-talk/cross-feeding events (Turroni et al., 2015; Turroni et al., 2018b).

Furthermore, a functional categorization of the up-regulated genes was performed using cluster of orthologous genes (COGs). Interestingly, both bi-and tri-associations of the assessed bifidobacterial strains caused a higher production of transcripts belonging to the amino acid and carbohydrate metabolism and transport families (COG-E and COG-G, respectively) (Figure 1C), unveiling a metabolism-biased response to the co-association (when compared to the mono-cultivation). Interestingly, while *B. longum* PRL2022, in all the tested conditions showed a similar number of up-regulated genes belonging to the metabolism-related COG families (Figure 1C), for *B. bifidum* PRL2010 the highest number of up-regulated genes related to the COG-E and COG-G families was recorded when in bi-association with *B. breve* PRL2012 and in the tri-association. Similarly, *B. breve* PRL2012 exhibited a higher number of up-regulated genes belonging to these COG families when co-cultivated with *B. bifidum* PRL2010 or in tri-association. These findings suggest that *B. longum* PRL2022 does not exhibit an affinity preference for a particular bifidobacterial species, as it interacts similarly from a metabolic point of view with each of the two tested species. In contrast, *B. bifidum* PRL2010 appears to exhibit an enhanced metabolic response when *B. breve* PRL2012 is present, and vice versa. Overall, despite differences in strain-specific gene modulation, these results indicate that these microorganisms, when exposed to particular (bifido) bacterial players, activate a range of metabolic options, ranging from cross-feeding events/cooperation to competitive behavior, to possibly improve their ecological fitness in a human gut-like environment. Furthermore, to assess whether co-associations among bifidobacterial strains led to growth differences compared to single strains, a qPCR assay was conducted using aliquots from the same samples on which RNAseq was performed. Notably, a statistically significant growth increase for *B. longum* PRL2022 was observed in both bi-associations and tri-association when compared to the mono-cultivation under the same conditions (Supplementary Table S11). Furthermore, even if only a tendency toward significance was observed, higher cell counts for *B. breve* PRL2012 were recorded when in bi-association with *B. longum* PRL2022 as well as for *B. breve* PRL2012 and *B. bifidum* PRL2010 in the case of tri-association and in bi-association with *B. longum* PRL2022 when compared to the mono-culture (Supplementary Table S11). Thus, suggesting a tendency toward better growth performances of the bifidobacterial strains when in bi-and tri-association when compared to mono-culture.

### Scrutiny of the co-association effect on the expression of bifidobacterial nutrient-related metabolic pathways

To further evaluate the impact that bifidobacterial co-cultivation may have on the modulation of metabolic pathways of a *Bifidobacterium* strain, an in-depth functional investigation into the observed up-regulated genes that belong to carbohydrate transport and metabolism (COG-G) was performed. Interestingly, the expression data revealed up-regulation of several genes encoding Glycosyl Hydrolases (GHs) and Glycosyl Transferases (GTs) in all bifidobacterial strains regardless of the co-association. Indeed, the higher abundance of transcripts related to genes involved in carbohydrate metabolism, as a consequence of co-cultivation with other microorganisms, may be considered a competitive response to advantageously access carbon sources, ensuring bifidobacterial growth and/or survival. On the other hand, it can also underline the establishment of cross-feeding events, which may contribute to promoting better metabolic fitness for all strains in co-association (Turroni et al., 2010a; Milani et al., 2014; Duranti et al., 2019; Luo et al., 2021; Diaz and Garrido, 2024; Supplementary Tables S2–S10). In detail, *B. breve* PRL2012, both in bi-and tri-association, produced a higher number of transcripts encoding for a predicted extracellular GH13 which acts as α-glucan-active enzyme (Turroni et al., 2016). Substrates of this GH13 include starch and related polysaccharides which are present both in the culture medium used here and are also widely available in the intestinal environment as nutrients internalized through the host diet (Alessandri et al., 2022; Fernandez-Julia et al., 2022; Table 1). Analysis of the *B. bifidum* PRL2010 transcriptome in bi-association with *B. breve* PRL2012 highlighted up-regulation of GH42, GH84, and GH33, i.e., enzymes predicted to be involved in mucin-associated O-/N-glycan degradation (Bell and Juge, 2021; Table 1). Specifically, while GH84 is an N-acetylglucosaminidase predicted to degrade the enteric mucin, the GH33 acts as an exo-sialidase capable of releasing sialic acid into the surrounding environment. In this context, since *B. breve* strains, unlike *B. bifidum* PRL2010, do not possess in their genomes exo-sialidases predicted to cleave mucin to release sialic acid, it is possible that the co-association of *B. bifidum* PRL2010 with *B. breve* PRL2012 specifically induced over-expression of genes involved in carbohydrate degradation/utilization to favor cross-feeding metabolic events, as has previously been described (Egan et al., 2014a; Egan et al., 2014b; Nishiyama et al., 2018; Turroni et al., 2018b; Yokoi et al., 2022). Therefore, this data suggests that the co-association of these bifidobacterial prototypes (when compared to the mono-cultivation) in a human gut-mimicking medium causes enhanced transcription of genes involved in the metabolism of both diet-and host-derived complex carbohydrates, thus enhancing their fitness by inducing cross-feeding interactions (Tannock et al., 2012; Egan et al., 2014a).

**Table 1:** List of up-regulated genes when *B. bifidum* PRL2010, *B. breve* PRL2012 and *B. longum* PRL2022 were in bi-association and tri-association in IGSM.

Species	Bi-association		Tri-association	Gene annotation
	*B. breve* PRL2012	*B. longum* PRL2022		
*B. bifidum* PRL2010	BBPR_RS00830	–	–	beta-galactosidase (GH42)
	BBPR_RS08290	–	–	beta-N-acetylglucosaminidase domain (GH84)
	BBPR_RS09085	–	–	exo-alpha-sialidase (GH33)
	BBPR_RS09090	–	–	exo-alpha-sialidase (GH33)
		–	BBPR_RS06710	1-deoxy-D-xylulose-5-phosphate synthase
		–	BBPR_RS02915	folylpolyglutamate synthase/dihydrofolate
	BBPR_RS01945	–	–	pyridoxal 5′-phosphate synthase glutaminase
	BBPR_RS09240	–	–	class C sortase (pili)
	BBPR_RS09250	–	–	LPXTG cell wall anchor domain-containing (pili)
	BBPR_RS08655	–	–	SpaA isopeptide-forming pilin-related protein (pili)
	BBPR_RS01460	–	–	isopeptide-forming domain-containing fimbrial (pili)
	BBPR_RS08910	BBPR_RS08910	BBPR_RS08910	DUF4244 domain-containing protein (pre-pilin Tad)
	BBPR_RS08920	BBPR_RS08920	BBPR_RS08920	pilus assembly protein (TadB)
	BBPR_RS08925	BBPR_RS08925	BBPR_RS08925	ATPase, T2SS/T4P/T4SS family (TadA)
	BBPR_RS08930	BBPR_RS08930	BBPR_RS08930	septum site-determining protein minD (TadZ)
	BBPR_RS08900	–	–	flp pilus-assembly TadE/G-like family protein
	BBPR_RS08905	–	–	TadE family type IV pilus minor pilin (TadE)
	*B. bifidum* PRL2010	*B. longum* PRL2022		
*B. breve* PRL2012	PRL2012_0103	PRL2012_0103	PRL2012_0103	glycoside hydrolase family 13 protein (GH13_30)
	PRL2012_0115	PRL2012_0115	PRL2012_0115	type I pullulanase (GH13)
	–	PRL2012_0617	–	sulfur carrier protein ThiS
	–	PRL2012_0620	–	rhodanese-like domain-containing protein
	PRL2012_1179	–	–	type I glyceraldehyde-3-phosphate dehydrogenase
	PRL2012_0107	PRL2012_0107	PRL2012_0107	class C sortase (pili)
	–	PRL2012_1610	–	SAF domain-containing protein (pili)
	–	PRL2012_1613	–	FHA domain-containing protein (pili)
	PRL2012_0174	PRL2012_0174	PRL2012_0174	GtrA family protein (EPS)
	PRL2012_0528	PRL2012_0528	PRL2012_0528	GtrA family protein (EPS)
	PRL2012_1735	PRL2012_1735	PRL2012_1735	polysaccharide ABC transporter ATP-binding (TA)
	PRL2012_1736	–	–	ABC transporter permease (TA)
	*B. bifidum* PRL2010	*B. breve* PRL2012		
*B. longum* PRL2022	67B_0509	67B_0509	67B_0509	1-deoxy-D-xylulose-5-phosphate synthase
	67B_0889	67B_0889	67B_0889	SufS family cysteine desulfurase
	67B_1137	67B_1137	67B_1137	transketolase
	67B_1138	67B_1138	67B_1138	transketolase
	67B_0815	67B_0815	67B_0815	pyridoxamine kinase
	67B_1943	67B_1943	67B_1943	ATP-binding cassette domain-containing protein (EPS)
	67B_1944	67B_1944	67B_1944	ABC transporter ATP-binding protein/permease (EPS)
	67B_0488	67B_0488	67B_0488	S-ribosylhomocysteine lyase (LuxS)

The first column specifies the bifidobacterial species used in the experiments. In the second column are listed all the up-regulated genes cited throughout the text in the bi-association for each bifidobacterial species. When not specified, the genes are up-regulated in both the bi-associations.

Beyond genes related to carbohydrate metabolism, it was also investigated whether the transcription of genes belonging to metabolic pathways involved in amino acid and coenzyme transport and metabolism (COG-E and COG-H families) underwent significant modifications for these bifidobacterial co-cultures. Interestingly, all three bifidobacterial strains, in every tested condition when compared to the mono-associations, showed transcriptional increase of several genes belonging to these COG families. Amino acids are fundamental for bifidobacterial growth and metabolism (Ventura et al., 2007). Additionally, since amino acid metabolism in bifidobacteria is closely associated with the production of metabolites (D’Aimmo et al., 2024; Dinger et al., 1991), including acetic acid, an in-depth assessment of the transcriptome associated with acetic acid metabolic pathway revealed a significantly higher number of transcripts of several genes in the three bifidobacterial prototypes regardless of the co-association (Supplementary Tables S2–S10). Thus, it can be inferred that co-association of these three bifidobacterial strains, compared to single mono-culture, promotes and stimulates metabolic pathways that may enhance the metabolic flux of the bifidobacterial strains (Arboleya et al., 2016; O'Callaghan and van Sinderen, 2016; Turroni et al., 2018a).

In addition, a detailed dissection of the bifidobacterial transcriptomes revealed the up-regulation of genes implicated in the biosynthesis of B vitamins. Specifically, *B. longum* PRL2022 in all the tested conditions, *B. bifidum* PRL2010 in the tri-association, and *B. breve* PRL2012 in the bi-association with *B. longum* PRL2022 unveiled the up-regulation of certain genes for thiamine production (vitamin B1) (Table 1). Interestingly, thiamine serves as a co-factor for pyruvate, an integral component of the metabolic pathway involved in the production of acetic acid (Soto-Martin et al., 2020). Since the latter may be used as a precursor to produce butyrate, i.e., a major short-chain fatty acid (SCFA) exerting numerous health benefits upon the host, including anti-inflammatory and anticarcinogenic effects (Louis et al., 2014), by butyrogenic members of the gut microbiota such as *Faecalibacterium prausnitzii*, this data suggests a beneficial cross-feeding interaction. Indeed, when co-cultivated with other intestinal players, bifidobacteria can indirectly contribute to the activity of butyrogenic gut microbiota members through this interaction (Gibson et al., 2016; Soto-Martin et al., 2020). Moreover, a gene involved in the biosynthesis of folate (vitamin B9) (Table 1), i.e., a vitamin linked to various physiological functions, including host immunity, gut barrier integrity, and even neurological health (Park et al., 2022), was shown to be up-regulated in *B. bifidum* PRL2010 in the tri-association. Additionally, the bi-association of *B. bifidum* PRL2010 and *B. breve* PRL2012 led to a significantly higher expression, in both strains, of genes involved in the biosynthesis of pyridoxine, along with *B. longum* PRL2022 in bi-and tri-associations (Table 1). Pyridoxine is crucial for the proper functioning of the metabolic pathways of bifidobacteria, participating in different pathways related to amino acid and carbohydrate metabolism, and regulating bifidobacterial growth and energy metabolism (Roberfroid et al., 2010; LeBlanc et al., 2013). Therefore, this data indicates that the co-cultivation of bifidobacterial strains is able to promote a higher expression of vitamin biosynthesis associated-genes, which may confer benefits to the host, compared to when the same bifidobacterial strains are individually cultivated.

### The impact of co-culture on bifidobacterial ability to interact with the host and other *Bifidobacterium* members

In order to evaluate whether co-association plays a role in enhancing bifidobacterial fitness in the human gut environment through genes other than those associated with metabolism, we performed an investigation into the genetic sequences that were shown to be up-regulated in the co-associations when compared to the mono-culture, and that were predicted to be involved in the interaction with the host and/or with other microbial players. In this context, a comprehensive analysis of the COG functional characterization revealed up-regulation of several gene clusters putatively involved in bifidobacterial interaction with other intestinal microorganisms or with the host (Supplementary Tables S2–S10). Specifically, all assessed co-associations caused up-regulation of a gene in *B. breve* PRL2012 predicted to belong to a sortase-dependent pilus locus (Table 1). Similarly, two genes of a homologous pilus locus were shown to be up-regulated in *B. bifidum* PRL2010 when in bi-association with *B. breve* PRL2012 as well as in the latter strain when co-cultivated with *B. longum* PRL2022 (Table 1). In this context, since sortase-dependent pili have mainly been depicted as extracellular structures directly involved in host–microbe interactions promoting adhesion to the human intestinal epithelial cells and aggregation between microbial cells, as well as cross-talk with the immune system (Turroni et al., 2013; Turroni et al., 2014a), these findings suggest that exposure of a bifidobacterial strain to another member of the genus *Bifidobacterium* plays a fundamental role in enhancing bifidobacterial ability to adhere to enterocytes. Therefore, this exposure favors their persistence in the human gut. Moreover, in all tested conditions, transcriptional up-regulation in *B. bifidum* PRL2010 of four genes belonging to the locus encoding for the Tad pilus, was observed (Table 1) (Alessandri et al., 2021). Particularly, enhanced transcription of *tad*Z, *tad*A (the ATPase) and *tad*B, involved in the formation and assembly of the pilus, was detected, along with the *flp* gene, which contributes to the production of the pilus structural proteins (O'Connell Motherway et al., 2011). Additionally, the presence of *B. breve* PRL2012, both in the bi-association with *B. bifidum* PRL2010 and in the tri-association, induced the up-regulation of two genes *tad*E also involved in the synthesis of the structural proteins of the pilus. In this context, the *tad* locus has been poorly characterized in bifidobacteria, except for *B. breve* UCC2003, and thus, the extracellular filament has been postulated to be involved in promoting adhesion of bifidobacteria to epithelial intestinal cells and cellular proliferation (O'Connell Motherway et al., 2011; Alessandri et al., 2021). Therefore, up-regulation of certain genes of the *tad* locus in *B. bifidum* PRL2010 indicates that co-association with other bacteria primes a cascade response aimed to produce extracellular structures to ensure its intestinal establishment and persistence. Similarly, beyond the significantly higher number of transcripts related to pilus production, in *B. breve* PRL2012 and *B. longum* PRL2022, both in bi-and tri-associations, an up-regulation of two genes implicated in EPS (exopolysaccharide) biosynthesis was observed (Table 1). Furthermore, in all tested conditions, *B. breve* PRL2012 was shown to exhibit an up-regulation of two genes which are predicted to play a role in the production of teichoic acids (Table 1). In this context, since both EPS and teichoic acids, i.e., glycan layers that cover the bacterial cell surface and negatively charged polymers present in/on the cell surface of Gram-positive bacteria, respectively, have been widely depicted as extracellular structures that participate in the interactions of bifidobacteria with the host or other bacterial players of the gut microbiota (Fanning et al., 2012; Alessandri et al., 2023; Argentini et al., 2023), these results strengthen the notion that bifidobacterial co-association stimulates the production of structures directly involved in bifidobacterial colonization of the intestine (Weidenmaier and Peschel, 2008; Colagiorgi et al., 2015; Rizzo et al., 2023).

Furthermore, the co-association of *B. longum* PRL2022 with the other two bifidobacterial strains led to enhanced expression of a gene predicted to encode the S-ribosylhomocysteine lyase (LuxS), i.e., the enzyme accountable for synthesizing autoinducer-2 (AI-2), along with the autoinducer-2 ABC transporter (Table 1). These two proteins contribute to the synthesis and transport of AI-2, triggering a complicated cell-to-cell communication system known as quorum sensing. Specifically, since AI-2 has been demonstrated to be involved in biofilm formation favoring bifidobacterial colonization of the gut ecosystem (Christiaen et al., 2014; Sun et al., 2014; Alessandri et al., 2023), the higher expression of *luxS* could be interpreted as evidence that the presence of *B. breve* PRL2012 and *B. bifidum* PRL2010 enhances the ability of *B. longum* PRL2022 not only to adhere to human host cells compared to when the strain is in mono-culture, but also to induce inter-microbial communication.

Altogether, these findings highlight that exposure of a single bifidobacterial strain to other members of the genus *Bifidobacterium* not only elicits a metabolism-biased response, but also triggers enhanced transcription of genes involved in the production of extracellular structures. Therefore, since the latter are known to play an important role in the establishment of a molecular dialog with the host, probably, the presence of other bacterial partner promotes in individual strains the establishment of a host-interacting bifidobacterial consortium. Clearly, more experiments with other bifidobacterial strains are needed to confirm this hypothesis.

### The molecular dialog of bifidobacterial tri-association with human cell monolayers

**Figure 2:**
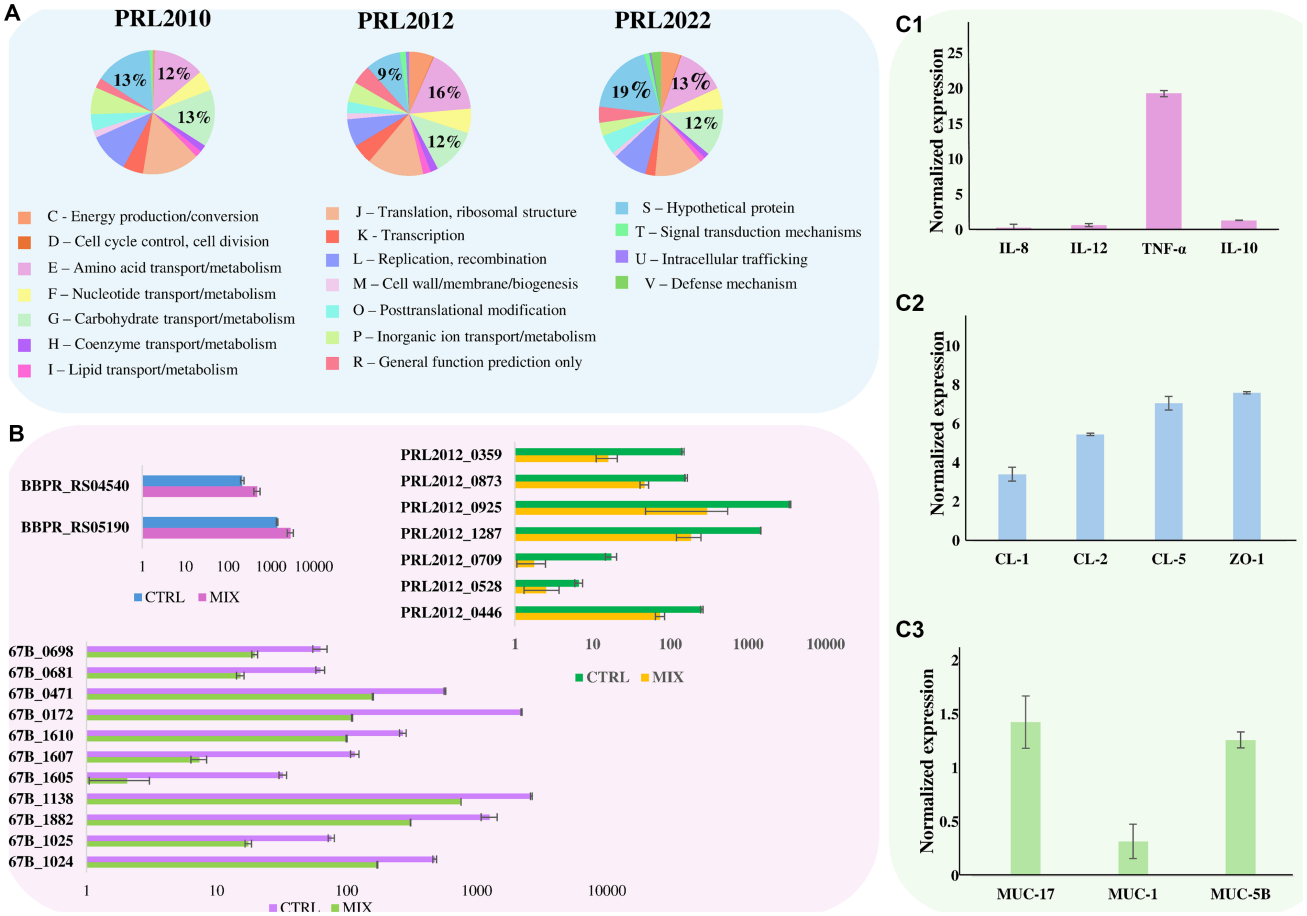
Effect of bifidobacterial tri-association exposure to human cell monolayers. Panel **(A)** depicts pie charts representing the percentage, calculated on the total number of overexpressed genes, of the number of up-regulated genes in *B. bifidum* PRL2010, *B. breve* PRL2012, and *B. longum* PRL2022, respectively, after being in contact for 4 h with a Caco-2/HT-29-MTX human cell monolayer of the tri-association when compared to the mono-association. Genes are divided according to the COG category they belong to. Panel **(B)** shows the transcriptional modulation of genes, cited in the text, of *B. bifidum* PRL2010, *B. breve* PRL2012, and *B. longum* PRL2022 after being in contact for 4 h with a Caco-2/HT-29-MTX human cell monolayer of the tri-association when compared to the mono-association. Transcriptional modulation of genes was reported as average of the normalized count reads obtained from each independent biological triplicate. Panel **(C1–C3)** represents the transcriptome levels of different genes of Caco-2/HT-29-MTX monolayer human cells after 4 h of incubation with the bifidobacterial tri-association. The y-axis represents the normalized expression level (ΔCt) according to CFX96 Bio-Rad software relative to the control (Caco-2/HT-29-MTX without bifidobacteria). The vertical bars indicate standard deviations.

The transcriptomic data originating from different combinations of bifidobacteria demonstrated that co-association of the three bifidobacterial prototypes caused transcriptional upregulation of a higher (compared to the mono−/bi-association) number of genes involved in host interaction, i.e., adhesion to the intestinal epithelia. To confirm the effect of increased up-regulation of genes mediating host interaction, *B. bifidum* PRL2010, *B. breve* PRL2012 and *B. longum* PRL2022 were cultured in tri-association on a Caco-2/HT-29-MTX cell monolayer for 4 h. Following this, differences in gene expression were evaluated through RNA sequencing between the bifidobacterial tri-associations in contact with human cell lines and the bifidobacterial mono-association, designated as bacterial control cells. Specifically, RNA sequencing generated a total of 10,110,619 quality-filtered reads with an average of 561,701 reads per sample (Supplementary Table S12). As described above, only genes showing a fold-change of ≥2 in combination with a *p*-value of ≤0.05 calculated through correction for multiple comparisons using the False Discovery Rate (FDR) procedure were considered as significantly differentially expressed between the two conditions. An in-depth investigation of the transcripts revealed a significant up-regulation of 126, 518, and 429 genes in *B. bifidum* PRL20120, *B. breve* PRL2012, and *B. longum* PRL2022, respectively, when in tri-association with respect to the mono-association (Supplementary Table S13). Particularly, a detailed investigation of the functional characterization of the up-regulated genes revealed that, in all three selected prototypes, the highest number of up-regulated genetic sequences in the tri-association, when compared to each mono-culture, belonged to carbohydrate and amino acid transport and metabolism COG families (Figure 2A). Interestingly, among the carbohydrate-utilization genes, several of the latter encode enzymes predicted to be secreted outside the bacterial cell. These enzymes, including various pullulanases, sialidases, and amylases, are involved in the cleavage of complex sugars that could increase their bioavailability for other bacteria. These findings confirm that also under conditions of direct contact with human intestinal cells, the tri-association among these three bifidobacterial strains induces a metabolic-biased response promoting the up-regulation of genes useful to promote a higher metabolic fitness of the bifidobacterial prototypes. Furthermore, in addition to genes involved in the metabolism and transport of carbohydrates and amino acids, several genes involved in bifidobacteria-host interaction underwent a significantly higher expression in the three prototypes when they were cultured on Caco-2/HT-29-MTX cell layers as tri-association with respect to mono-associations. Interestingly, in *B. breve* PRL2012 up-regulation of four genes belonging to different loci involved in EPS biosynthesis was observed (Table 2; Figure 2B). Similarly, three genes belonging to two loci for EPS assembly displayed significantly higher levels of expression in *B. longum* PRL2022 (Supplementary Table S13). Moreover, genes encoding for transaldolases were detected in all three bifidobacterial strains when placed in contact with human cell lines as tri-association (Table 2; Figure 2B). In this context, since both EPS and transaldolases have been identified as extracellular structures that facilitate attachment to the mucosal surface of the host, it can be inferred that exposure to other bacteria may have a role in activating strategies to enhance bifidobacterial binding capabilities to the intestinal epithelial surface (Nishiyama et al., 2020; Shang et al., 2022). Additionally, the tri-association induced in *B. longum* PRL2022 the up-regulation of two genes encoding two fibronectin type III domain containing-proteins as well as a higher number of transcripts encoding a GH136 (Table 2; Figure 2B). In detail, since proteins harboring a fibronectin type III domain have been shown to promote anchoring to the extracellular matrix protein and the GH136 has been described as a mucin-degrading glycosyl hydrolase also implicated in bifidobacterial gut persistence, this data strengthens the notion that the tri-association promotes the up-regulation of genes responsible for microbe-host interaction to improve strain fitness in the human intestine and favoring their persistence (Yamada et al., 2017; van Leeuwen et al., 2021; Alessandri et al., 2023; Rizzo et al., 2024). Notably, *B. bifidum* PRL2010 did not show a significant increase in transcripts corresponding to genes involved in the production of extracellular structures when the tri-association seeded on Caco-2/HT-29-MTX cell line monolayer was compared to the mono-association. However, upon analyzing the transcriptomic data, it was observed that these genes were highly expressed in both mono-and tri-association after the exposure to human intestinal epithelial cells, thus suggesting that the production of these extracellular structures is more likely due in response to the contact with the human cell lines regardless of the interaction with other bifidobacteria. Furthermore, beyond those genes aimed at interacting with the host, the tri-association stimulated the up-regulation of genes involved in mucin degradation, i.e., an N-acetylgalactosaminidase in both *B. breve* PRL2012 and *B. longum* PRL2022 (GH129 and GH101, respectively), along with the induction of a mucin glycan degradation enzyme, i.e., GH42, in both bifidobacterial strains (Table 2; Figure 2B). In this context, since the HT-29-MTX is a mucin-secreting cell line (Bianchi et al., 2019), the bifidobacterial tri-association may have favored a significantly higher production of transcripts corresponding to enzymes that utilize mucin as a carbon source. This is a prerogative of a restricted set of intestinal bacteria, which provides bifidobacteria with a selective advantage in a competitive environment such as the intestine, ensuring their growth, proliferation and persistence (Gotoh et al., 2023; Alessandri et al., 2021; Egan et al., 2016; Katoh et al., 2017; Katoh et al., 2020).

**Table 2:** List of up-regulated genes when *B. bifidum* PRL2010, *B. breve* PRL2012 and *B. longum* PRL2022 were in contact in tri-association with Caco-2/HT-29-MTX.

Species	Tri-association	Gene annotation
*B. bifidum* PRL2010	BBPR_RS05190	Transaldolase
	BBPR_RS04540	Type II toxin-antitoxin system HicB
*B. breve* PRL2012	PRL2012_0446	Amino acid ABC transporter ATP-binding (EPS)
	PRL2012_0528	GtrA family protein (EPS)
	PRL2012_0709	IS3 family transposase (EPS)
	PRL2012_1287	amino acid ABC transporter ATP-binding (EPS)
	PRL2012_0925	transaldolase
	PRL2012_0873	glycoside hydrolase (GH129)
	PRL2012_0359	beta-galactosidase (GH42)
*B. longum* PRL2022	67B_1,024	glycogen synthase (EPS)
	67B_1025	ATP-binding cassette domain-containing (EPS)
	67B_1882	ATP-binding cassette domain-containing (EPS)
	67B_1138	Transaldolase
	67B_1,605	Glycoside hydrolase family 3\u00B0C-terminal (GH3)
	67B_1607	Glycoside hydrolase family 3\u00B0C-terminal (GH3)
	67B_1610	Right-handed parallel beta-helix repeat (GH136)
	67B_0172	endo-alpha-N-acetylgalactosaminidase(GH101)
	67B_0471	beta-galactosidase (GH42)
	67B_0681	type IV toxin-antitoxin system AbiEi
	67B_0698	type II toxin-antitoxin system RelB/DinJ

The first column specifies the bifidobacterial species used in the experiments. In the second column are listed all the up-regulated genes cited throughout the text in the tri-association for every bifidobacterial species.

In addition, it is worth noting that the tri-association favored transcriptional up-regulation of genes involved in the toxin-antitoxin system in *B. bifidum* PRL2010 and *B. longum* PRL2022 when they were in contact with human cell lines (Table 2; Figure 2B). In this context, since the toxin-antitoxin system is implicated in bacterial persistence, biofilm formation, and antibiotic tolerance (Klimina et al., 2019; Klimina et al., 2020), the observed up-regulation of the two genes constituting this system may be considered as an additional strategy to improve bifidobacterial survival in the human intestine.

Finally, a notable number of up-regulated genes belonging to the category of genes whose function is unknown (COG-S) were detected in all three bifidobacterial strains when in contact, as a tri-association, with human cell monolayers (Figure 2A). In this context, the significantly higher number of transcripts corresponding to genes encoding proteins with unknown functions in the three bifidobacterial prototypes when they are in contact with human cell lines as a tri-association, not only underlines that a plethora of yet-to-be-characterized proteins may play a role in enhancing bifidobacterial persistence in the human gut by improving their dialog with host cells as well as by promoting their interaction with the host and other bacterial players. It also emphasizes the need to characterize these bifidobacterial proteins of unknown function to gain in-depth insight into the strategies that bifidobacteria may use and activate to interact with the host and other bacteria, ensuring their persistence in the human gut.

Overall, these data suggest that the association of the three bifidobacterial prototypes induces activation of various strategies that bifidobacteria may exploit to successfully interact with the human intestinal epithelium, ensuring their persistence within the human intestinal environment.

### Evaluation of host response after exposure to bifidobacterial tri-association

To evaluate whether and how the interaction between the bifidobacterial co-culture and the Caco-2/HT-29-MTX cell monolayer modulates the gene expression not only in the bacterial cells but also in the eukaryotic cells, RNA extracted from the human cell line after exposure to bifidobacterial tri-association was analyzed through the evaluation of the induction of specific sets of genes through RT-PCR. Caco-2/HT-29-MTX cell monolayers not exposed to any bifidobacterial strains were used as controls. Specifically, genes encoding different cytokines were analyzed to assess if and how these human cells may respond from an inflammatory perspective when placed in contact with bifidobacterial strains when compared to the absence of any microbial cells. Additionally, genes responsible for maintaining the integrity and homeostasis of the intestinal epithelial barrier, including genes coding for tight junction proteins as well as genetic sequences involved in mucous layer production, were also examined. Interestingly, contact between the bifidobacterial tri-association and the human cell monolayer revealed higher levels of tumor necrosis factor (TNF)-α mRNA, a mild induction of the anti-inflammatory interleukin (IL)-10 and a lower induction of the pro-inflammatory IL-12 and IL-8 response compared to the human cells not exposed to bifidobacteria (Figure 2C1). This finding supports previous reports; indeed, bifidobacteria have been described as strong stimulators of TNF-α but less effective inducers of other pro-inflammatory cytokines, typically associated with systemic immune responses (Fink et al., 2007; Okada et al., 2009; Weiss et al., 2010). In fact, the IL-10/IL-12 ratio (ratio of 1.97) suggests a higher induction of the anti-inflammatory cytokines with respect to the IL-12 specifically involved in a systemic inflammatory response (Hildenbrand et al., 2023). These results suggest that bifidobacterial tri-association plays a role in initiating communication among immune cells without a system triggering inflammation, instead, alerting the immune system to quickly respond to potential pathogens (Latvala et al., 2011; Fanning et al., 2012; Turroni et al., 2013; Turroni et al., 2014b). Moreover, different genes involved in the regulation of the homeostasis and integrity of the intestinal epithelial barrier were substantially up-regulated when compared to the unexposed control (Pope et al., 2014; Kuo et al., 2021; Ahmad et al., 2023; Figure 2C2). This indicates that the bifidobacterial tri-association may induce beneficial effects upon the human host contributing to the reinforcement of the intestinal barrier.

Additionally, the MUC5B and MUC17 genes which encode for a mucin-related epithelial glycoprotein and a major gel-forming mucin protein, respectively, were also slightly over-expressed (gene expression of 1.26 and 1.43, respectively) in the Caco-2/HT-29-MTX cell layer in contact with the bifidobacterial cells, with respect to the human cells without bifidobacterial association (Figure 2C3). Instead, the MUC1 gene showed an opposite trend (gene expression of 0.32), displaying a down-regulation compared to the control. This gene encodes a transmembrane glycoprotein abnormally over-expressed in colorectal carcinoma also associated with venous thrombosis in cancer patients (Guo et al., 2018; Szlendak et al., 2020; Kawano et al., 2024). The observed down-regulation of MUC1 may open a potential avenue for studying the impact that bifidobacteria might have on limiting/reducing the likelihood of tumor onset and possible complications. Thus, these findings showed that the bifidobacterial co-association activated the up-regulation of genes involved in mucin degradation, which may indeed be a stimulus for mucin production by eukaryotic cells, potentially aiding in strengthening the integrity of the intestinal barrier along with the down-regulation of a gene involved in the onset of colorectal cancer. Clearly, although these data suggest potential beneficial effects of bifidobacteria toward intestinal health, since the eukaryotic-prokaryotic cell interactions are deeply complicated and depend on multiple factors, other experiments aimed at characterizing the molecular aspects behind these interactions are necessary.

However, these data confirm the hypothesis that the bifidobacterial co-association with the human intestinal cells contributes to the enhancement of the epithelial barrier, thereby potentially promoting health benefits for the host.

## Conclusion

Although bifidobacteria are common residents in the human gut and promote health, few studies evaluate the impact of their co-association on both bacteria and host. To explore potential synergistic interactions, we compared the transcriptomes of bi-and tri-associations of *B. bifidum* PRL2010, *B. breve* PRL2012, and *B. longum* PRL2022 to monocultures. Co-association enhanced gene transcription related to carbohydrate, amino acid, and vitamin metabolism and transport, suggesting improved energy harvest and cross-feeding activities. Additionally, the tri-association up-regulated genes for producing extracellular structures like pili, EPS, and teichoic acids, enhancing the bacterial ability to adhere to and interact with the host. Transcriptomic data from tri-associations on Caco-2/HT-29-MTX cells showed higher expression of genes for extracellular structures and mucin degradation, a potential for increased adherence and persistence in the intestine and activation of carbohydrate-degrading activities for growth. Gene expression analyses on human cells in contact with the tri-association revealed changes, particularly in inflammatory response genes. The tri-association did not induce systemic inflammation but stimulated a localized response, pointing out the potential that this interaction may have on educating the immune system for a rapid response against potential pathogens, suggesting that further experiments would be useful to demonstrate this hypothesis. This study demonstrates the transcriptional response of the tri-association, which may be associated with increased survival, metabolism, growth, and persistence in a simulated healthy intestinal environment.

## Data Availability

Raw sequences of RNA sequencing data are available in the SRA database with accession number PRJNA1108206.
